# Social radiology: Where to now?

**DOI:** 10.2349/biij.8.1.e9

**Published:** 2012-01-01

**Authors:** ELM Ho

**Affiliations:** Imaging Department, Sime Darby Specialist Centre Megah Sdn. Bhd., Kuala Lumpur, Malaysia.

**Keywords:** Radiology services, diagnostic imaging, social radiology, radiation protection and safety, outreach programmes, developing countries

## Abstract

Radiology is a relatively high-cost and high-maintenance aspect of medicine. Expertise is constantly required, from acquisition to its use and quality assurance programmes. However, it is an integral part of healthcare practice, from disease diagnosis, surveillance and prevention to treatment monitoring. It is alarming that two thirds of the world is deficient in or lacks even basic diagnostic imaging. Developing and underdeveloped countries need help in improving medical imaging. Help is coming from various organisations, which are extending hands-on teaching and imparting knowledge, as well as training trainers to increase the pool of skilled practitioners in the use of imaging equipment and other aspects of radiology services. The scene for social radiology is changing and set to positively impact the world in the (near) future.

## SOCIAL RADIOLOGY: WHERE TO NOW?

The word “social” here refers to organised efforts to advance human welfare, normally directed or designed to help the poor, aged, or young. In war-torn countries or countries afflicted with calamities such as disease epidemics, earthquakes, tsunamis or drought, the first thing that springs to mind is medical aid. Medical aid organisations such as Medecins Sans Frontieres (MSF) are well known for efforts to deliver emergency aid in many such countries. Such work of course comes with great risks, especially in politically unstable countries or in a war [1].

Radiology today includes diagnostic imaging, image-guided intervention, and monitoring treatment response. It comes at a relatively high cost and is resource-hungry, requiring infrastructure as well as expertise in its use, from purchasing to maintenance and quality control. It is no longer the exclusive domain of radiologists, but of trained practitioners skilled in its use for maximal impact on healthcare. When it entails ionising radiation, there are additional issues of radiation protection and safety [2].

There are many resource-limited areas where healthcare is deficient and where radiology services are lacking. The World Health Organisation has recognised that basic radiology services such as X-ray and ultrasound imaging are vital in the surveillance, prevention, and diagnosis of diseases, as well as in monitoring treatment [3]. Yet two thirds of the world lacks such basic diagnostic radiology services. Even when equipment is available, it may be mismatched with requirements or operational expertise, or it may not be maintained properly or used safely. This has resulted in relatively few social radiology service provision programmes, compared to other social medical services, such as the distribution of vaccines and antimalarial drugs.

Outreach programmes, especially educational programmes, are not new in radiology. The Radiological Society of North America (RSNA) has outreach programmes that have been operational for many years [4]. Malaysia, being a developing country, has benefitted from the RSNA International Visiting Professor Programme, most recently in 2011. The American College of Radiology has online resources that are available to those in developing countries [5].

In the last few years, outreach programmes have gained another dimension. Actual train-the-trainers and train-the-users programmes in remote areas, especially in Africa, have begun. The Physicians Ultrasound in Rwanda Education (PURE) initiative was born in 2010 out of a need to train physicians to operate donated ultrasound machines lying unused in hospitals in Rwanda. PURE was founded by emergency physicians in the United States of America and now has volunteers from other countries [6].

Ultrasound as an imaging modality is radiation-free and its portability makes it immensely useful in many acute medical conditions as well as in maternal and child health. However, it still requires a stable electricity source and great skill in the use and interpretation of the findings. The skill set must be tailored to the common conditions where the service is to be integrated into the healthcare system. It is therefore also no surprise that the International Society of Ultrasound in Obstetrics and Gynaecology (ISUOG) also has an outreach programme in ultrasound training for underserved areas [7]. Their first outreach project was conducted in Manila in 1996. Intensive hands-on training is only one aspect of the “technology transfer” but they also provide the essential knowledge behind ultrasound so that it can be used more masterfully and appropriately. There is also an emphasis on continuing education and training. Valuable lessons learnt over the years from “teaching-the-teachers” include the need to be culturally sensitive, to adjust training to local needs, to nurture communication and relationships with those involved as well as university leaders, and – crucially – to obtain feedback from the trainees. These have included midwives, physicians, and radiographers. They may partner a local university hospital, MSF, and a generous vendor that donates the ultrasound equipment. An example is their effort in Somaliland.

However, ultrasound is not the only social service provision that is feasible. RAD-AID International is now emerging as a force that may indeed change the face of social radiology positively and remarkably [8]. Founded by a team of radiologists at Johns Hopkins School of Medicine, RAD-AID is a dynamic non-profit organisation that is determined to help developing countries implement and optimise radiology and health imaging services whilst ensuring these radiology services integrate with public health programmes. RAD-AID partners many other organisations, some experienced in the provision of humanitarian medical aid while others are involved in developing cheaper yet robust ways to deliver modern radiology services. They have run a lectureship exchange programme in China since 2010 and are building a new women’s health mobile clinic for North India. Their free annual conference on international radiology for developing and emerging countries has been extremely successful. Sustainable strategies have been identified, including financing models, donor education, practitioner education, public health efforts, technology innovation and implementation, as well as sustainable clinical models [9]. The conference provides a platform for all stakeholders interested in improving and developing systems for medical imaging where they are lacking. Slide decks from some of the conference presentations are available on the RAD-AID website.

Digitisation may be a boon with respect to access and availability. Cloud Picture and Archiving Systems (PACS) may obviate the financial burden of ownership costs. Digitisation has also removed the need for the safe disposal of processing chemicals. Teleradiology allows the globalisation of expertise, although the lack of information and communications technology infrastructure in remote areas still needs to be overcome.

Challenging needs demand innovative solutions. The scene is definitely changing for radiology. Social radiology is poised to play a greater role in helping developing and underdeveloped countries improve their radiology/imaging services as an integral part of their public health systems. Now is the time for more organisations of the non-acute version of “Radiology Sans Frontieres” to come to the fore – or would that be wishful thinking?
